# Metabolic and redox signaling in the retina

**DOI:** 10.1007/s00018-016-2318-7

**Published:** 2016-08-20

**Authors:** Thierry Léveillard, José-Alain Sahel

**Affiliations:** 1 Department of Genetics, Institut de la Vision, Sorbonne Universités, UPMC Univ Paris 06 UMR_S968, INSERM_U968, CNRS UMR_7210, 17 rue Moreau, 75012 Paris, France; 2 CHNO des Quinze-Vingts, DHU Sight Restore, INSERM-DGOS CIC_1423, 28 rue de Charenton, 75012 Paris, France

**Keywords:** Cone photoreceptor, Retinal degeneration, Aerobic glycolysis, Glucose transporter, Thioredoxin, Pentose phosphate pathway, Nucleoredoxin-like genes, Rod-derived cone viability factor

## Abstract

Visual perception by photoreceptors relies on the interaction of incident photons from light with a derivative of vitamin A that is covalently linked to an opsin molecule located in a special subcellular structure, the photoreceptor outer segment. The photochemical reaction produced by the photon is optimal when the opsin molecule, a seven-transmembrane protein, is embedded in a lipid bilayer of optimal fluidity. This is achieved in vertebrate photoreceptors by a high proportion of lipids made with polyunsaturated fatty acids, which have the detrimental property of being oxidized and damaged by light. Photoreceptors cannot divide, but regenerate their outer segments. This is an enormous energetic challenge that explains why photoreceptors metabolize glucose through aerobic glycolysis, as cancer cells do. Uptaken glucose produces metabolites to renew that outer segment as well as reducing power through the pentose phosphate pathway to protect photoreceptors against oxidative damage.

## Upside down: considerations of the inverted camera type eye

**Fig. 1:**
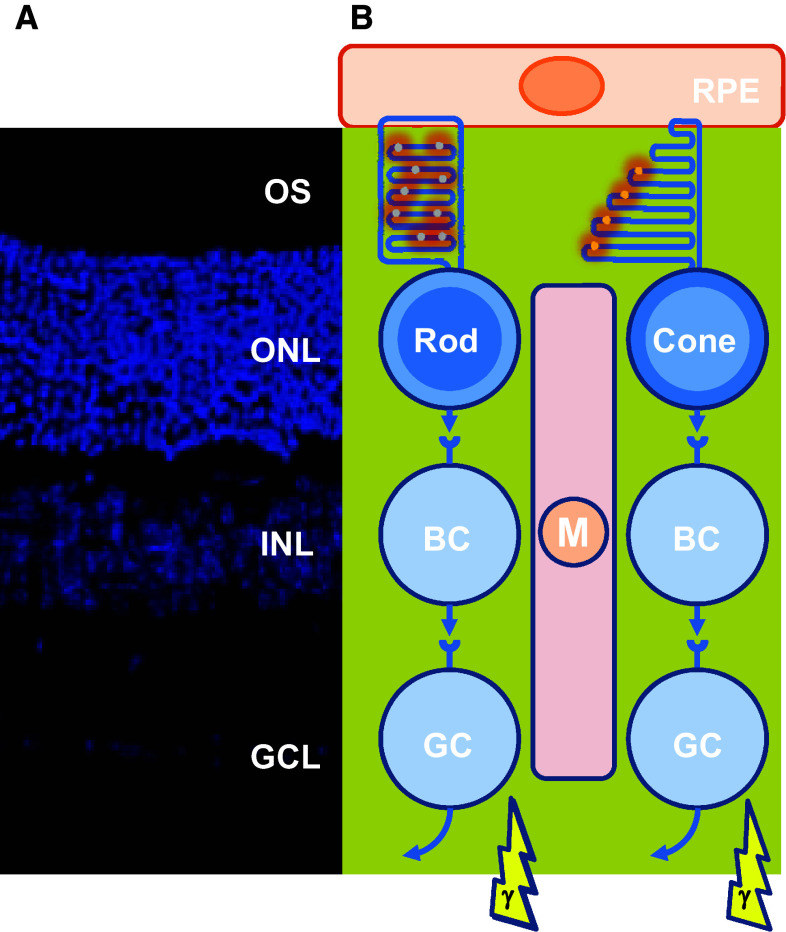
Architecture of the retina of vertebrates. **a** Mouse adult retinal section with nuclei labeled with 4′,6-diamidino-2-phenylindole (DAPI). *OS* outers segment, *ONL* outer nuclear layer, *INL* inner nuclear layer, *GCL* ganglion cell layer. **b** Schematic drawing of the retinal cells and their functional relations. *RPE* retinal pigmented epithelium, *BC* bipolar cell, *GC* ganglion cell, *M* Muller glial cell

**Fig. 2:**
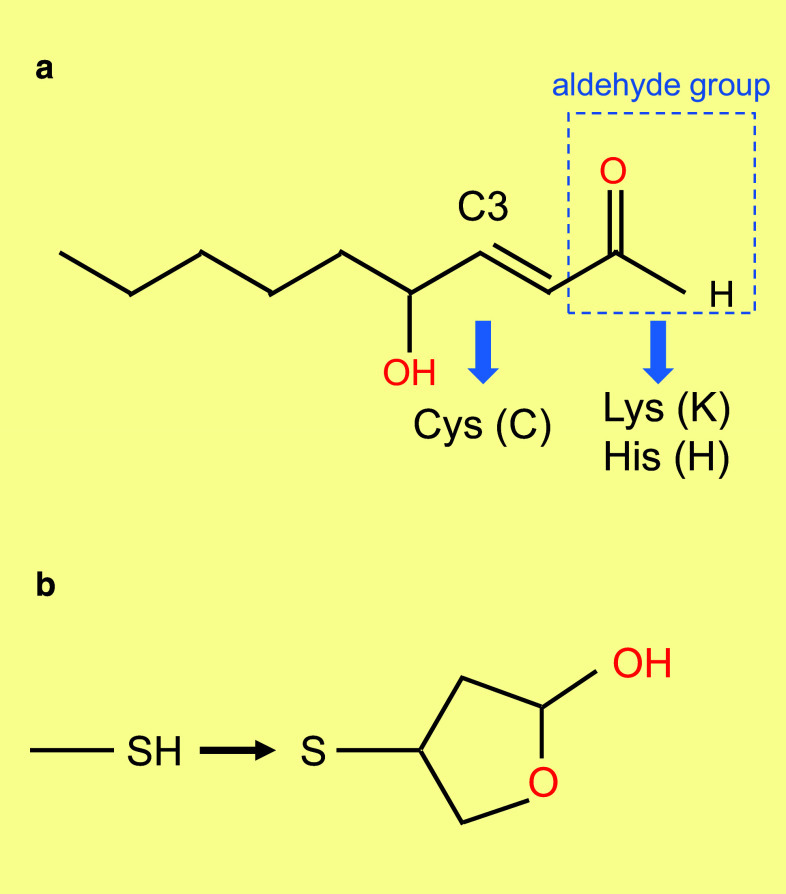
Lipid peroxidation chain reaction. **a** Chemical structure of 4-hydroxy-2-nonenal (HNE). The carbon at position C3 targets cysteine modification. The aldehydes group targets lysine and histidine modifications. **b** Modification of a cysteine residue (SH) in a protein by HNE through thiol Michael addition at position C3

The vertebrate retina, the light sensitive part of the eye, is composed of three layers of neurons and of radial Muller glial cells. The photoreceptor layer, which includes rods and cones, is located in the farthest position with respect to the incidence of light; their nuclei form what we call the outer nuclear layer as observed on retinal sections (Fig. 1). The other layers are composed of interneurons, such as bipolar cells that relay light-dependent electrochemical signals, transmitted through the photoreceptor synapses, to neurons of the ganglion cell layer. The axons of these later neurons form the optic nerve. Within the circuit, other neurons intercalated into the retina modulate the signal. The biological rational of this counterintuitive optic setting is explained by the chemical properties of the photoreceptor cellular substructure that captures the photon, the photoreceptor outer segment [1]. Most engineers would place the photoreceptors of the retina to the nearest from the incident light. The high sensitivity of the retina requires that the number of light sensing molecules, the opsins, be very high. Both for vertebrates and invertebrates, the opsin molecules are seven-transmembrane domains proteins coupled to G protein, alternatively named G protein-coupled receptors. Opsins establish a covalent link through an intra-membranal lysine residue with a chromophore derived from vitamin A. Retinal exists as two stereoisomers, 11-*cis*-retinal and all-*trans*-retinal. Upon absorption in the visible range, a photon triggers *cis*–*trans* isomerization; the chromophore is converted from a bent molecule to a straight one by the energy provided to pass the thermodynamic barrier separating the two stereoisomers. The straightening of the chromophore within the hydrophobic pocket formed by the seven-transmembrane α-helix induces a conformational change that is sensed by an intra-cellular G protein, the transducin. This molecular rearrangement is optimal within a lipid bilayer of high fluidity [2]. The fluidity of the lipid bilayer of the photoreceptor outer segment is conferred by its high proportion of lipids made of polyunsaturated fatty acids (PUFA). In mammals, docosahexaenoic acid (DHA, 22:6, n-3), an essential omega-3 fatty acid, accounts for 80 % of the PUFAs of photoreceptor outer segment. Polyunsaturation is the existence of several double bonds (C=C), which are chemically rigid. Nevertheless, C=C bonds of PUFA are flanked by two saturated bonds (C–C) forming a regular pattern of one non-rotating (C=C) and two rotating bonds (C–C). This arrangement reduces the energy of rotation that increases the fluidity of the lipid membrane [3]. It is also a double bond (C=C) of the chromophore that is subject to *cis*–*trans* isomerization. Lipids of the photoreceptor outer segment are prone to oxidation. Reactive oxygen species (ROS) drive lipid peroxidation, a chain reaction, in which one ROS can induce the oxidation of a large number of lipid molecules-containing PUFA [4]. Fatty acid hydroperoxides are finally decomposed into reactive aldehydes, such as 4-hydroxy-2-nonenal (HNE) and malondialdehyde (MDA). Monounsaturated and saturated fatty acids are much less reactive and do not usually participate in lipid peroxidation. The end-products of lipid peroxidation (MDA and HNE) cause protein damage by reacting with chemical groups within certain amino acids as cysteines, lysines, and histidines [5] (Fig. 2a). The nucleophilic thiol side chain in cysteine participates in many enzymatic reactions and the irreversible formation of HNE adduct with photoreceptor proteins is detrimental to their function [6] (Fig. 2b). Photoreceptors are post-mitotic neurons that do not regenerate, at least in mammals. The damaged lipids are eliminated from vertebrate photoreceptors by phagocytosis of disks by the retinal pigmented epithelium (RPE). This process is regulated by the circadian clock, so that 10 % of rod photoreceptor outer segment is daily engulfed in phagosomes of RPE cells. Phagosomes are moved from the apical membrane toward the basal membrane where their contained in proteins and lipids are degraded [7]. To maintain its length, the photoreceptor outer segment is renewed from its base, a process that involves protein and lipid synthesis in the inner segment of photoreceptor, a cellular substructure just beneath the outer segment. Contrarily to the cones, the rod outer segments are made up of a stack of individualized membranal disks unconnected to the plasma membrane of the inner segment. Consequently, lipids are transferred from the plasma membrane to the disks [8–10].

The renewal of rod outer segments was elegantly discovered by Young [11]. In rods, most of the new proteins are first concentrated at their base, where they are used in the assembly of new disk membranes. After a single injection of ^3^H-methionine, he observed by autoradiography, labeled disks progressively displaced along the outer segment due to the repeated formation of newer disks. A similar observation was made using tritium-labeled fatty acids [12]. Most likely, it is this mechanism of disk shedding and renewal that imposed the upside down positioning of the photoreceptors and their outer segments in the most distal part of the retina from the incident light. One could argue that this is not the only possible organization of photoreceptors in the eye, since cephalopods have an everted retina, so that the distal end of rhabdomeric photoreceptors is pointing toward incoming light [13].

## The life of photoreceptors: a challenging task

**Fig. 3:**
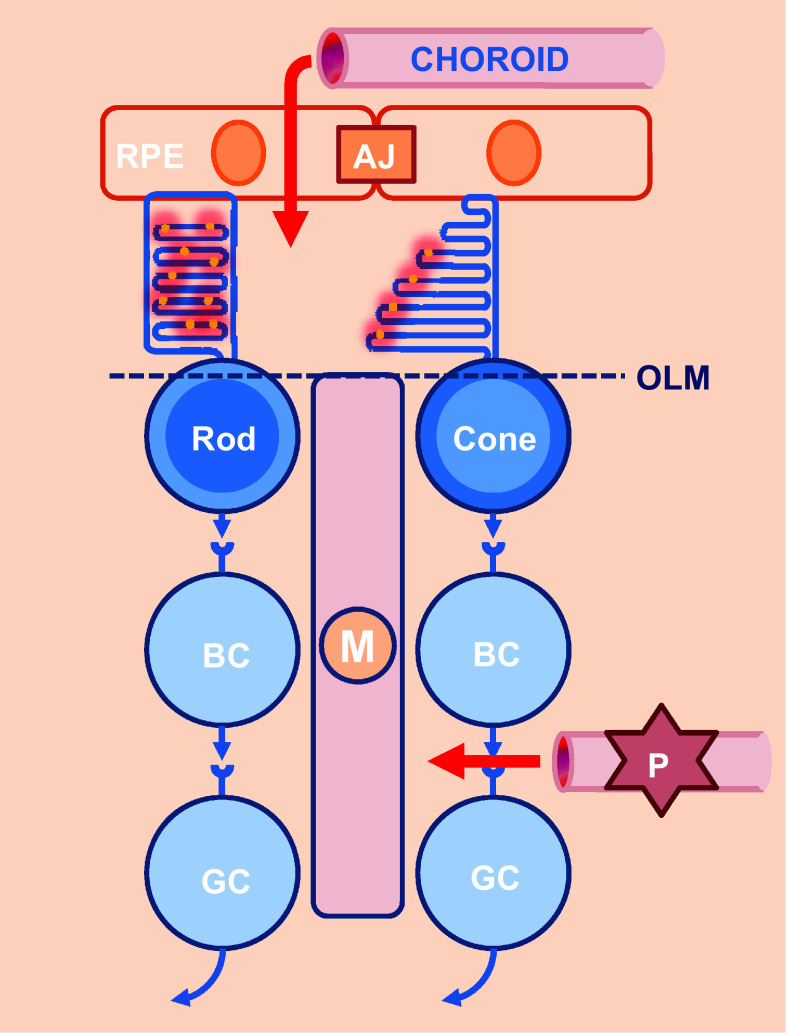
Exchanges between the retina and the blood circulation. Blood circulation is controlled at two levels (*red arrows*): a blood-retinal barrier in the inner retina and an outer retinal barrier, which is constituted by the RPE. *RPE* retinal pigmented epithelium, *BC* bipolar cell, *GC* ganglion cell, *M* Muller glial cell, *AJ* adherent junction, *OLM* outer limiting membrane, *P* pericyte

The renewal of photoreceptor outer segment in vertebrate retina is energetically demanding and biologically challenging. The outer retina formed by photoreceptors is avascular, in contrast to the inner retina. Exchanges between the retina and the circulation are controlled at two levels: the blood-retinal barrier in the inner retina, made up of retinal vessels surrounded by pericytes and glial cells, and the outer retinal barrier, which is constituted by the RPE adherent junctions (Fig. 3). All nutrients, including glucose, vitamin A, and fatty acids, are provided to photoreceptors by choroidal blood flow behind the RPE, one of the highest rates of blood flow of the whole body [14]. The blood supply to photoreceptors must be transported through RPE cells that form an epithelium with adherent junctions.

## Good fats: supplying fatty acids to photoreceptor cells

**Fig. 4:**
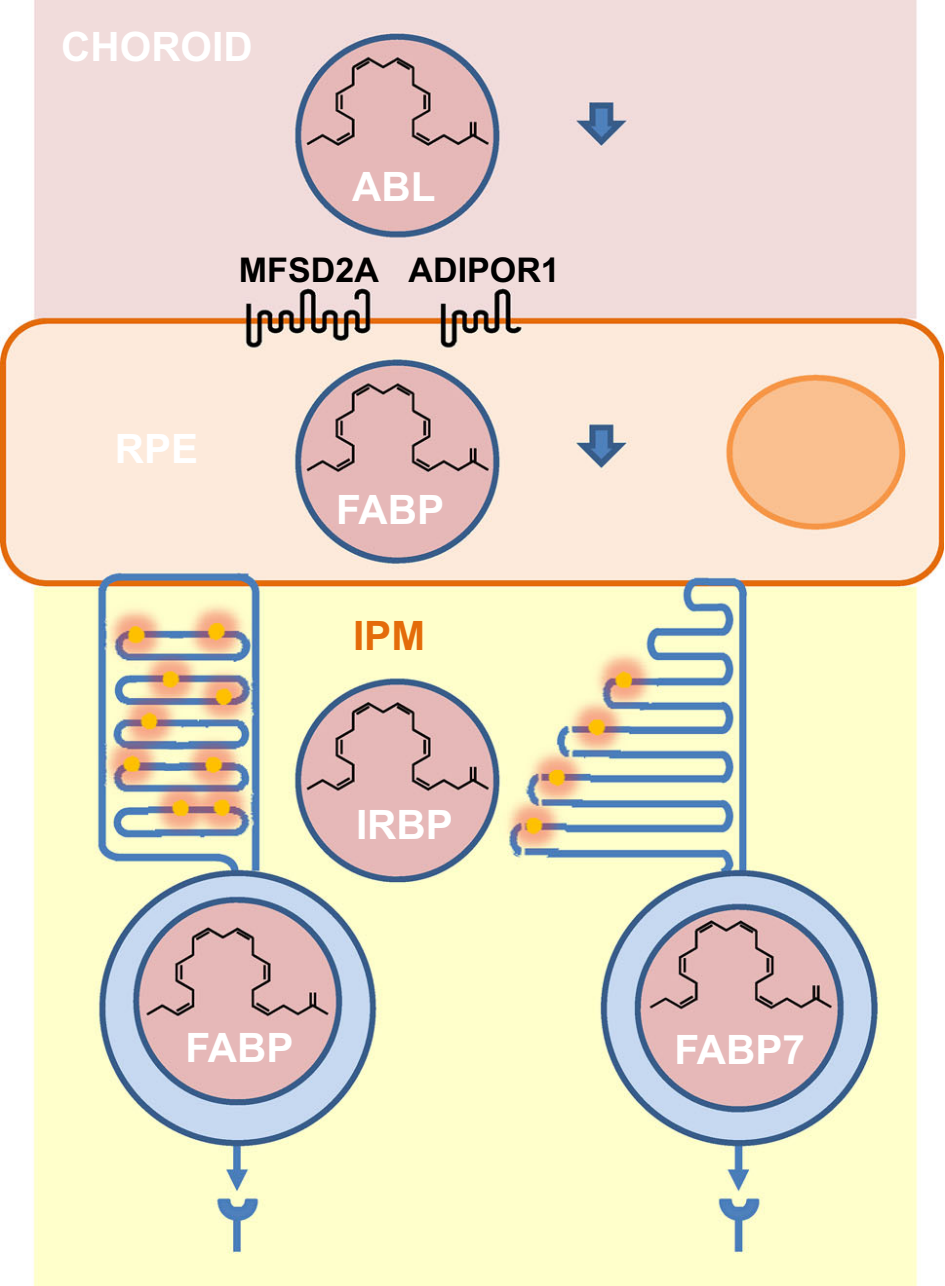
Transport of essential fatty acids from the blood circulation to photoreceptors. *RPE* retinal pigmented epithelium, *ABL* albumin, *ADIPOR1* adiponectin receptor 1, *MFSD2A* fatty acid transporter, *IPM* inter-photoreceptor matrix, *FABP* fatty acid-binding protein, *IRBP* inter-photoreceptor retinoid-binding protein

It is broadly accepted that in mammals, unsaturated fatty acids are not synthesized in the retina but originate from blood supply. The essential fatty acids, among which DHA (C22:6, n-3) and its precursor, α-linolenic acid (18:3, n-3) are hydrophobic molecules transported by serum albumin from the liver to the basal side of the RPE cells where they are transferred to photoreceptors through the apical side (Fig. 4). Two distinct molecules have been implicated in the transport of DHA through the RPE, the major facilitator superfamily domain-containing protein 2a (MFSD2A) and the adiponectin receptor 1 (ADIPOR1) [15–17]. MFSD2A is a typical 12 transmembrane domains transporter, while ADIPOR1 is an atypical 7 transmembrane domains receptor of adiponectin, an essential hormone secreted by adipocytes that regulates glucose and fatty acid metabolism. The mechanisms that link the two transporter proteins are unknown.

Transport of insoluble fatty acids from basal to apical surfaces of the RPE certainly involves transient interactions with fatty acid-binding proteins (FABP) to allow intra-cellular translocation of hydrophobic molecules in the aqueous cytosol.

Essential fatty acids as DHA retrieved from shed photoreceptor apical disk membranes are recycled back to the photoreceptor inner segment and further incorporated into phospholipids of renewed photoreceptor outer segments. The recycled fatty acids are transferred from the RPE to photoreceptors through the inter-photoreceptor matrix (IPM), in the extracellular space between the photoreceptor outer segments and the RPE, in the absence of albumin, the inter-photoreceptor retinoid-binding protein (IRBP) binds to fatty acids [18, 19]. Since the expression of MFSD2A by photoreceptors was not reported, it is still unclear how essential fatty acids are taken up by photoreceptors, but pulsed-labeling experiments with radiotracers demonstrated that this happens in vitro and in vivo [20]. To be incorporated into the renewing photoreceptor outer segments, non-polar fatty or aliphatic acid tails part of the amphiphilic phospholipid must be linked to a polar head derived from glycerol-3-phosphate for glycerophospholipids [21]. This is taking place at the surface of the endoplasmic reticulum in the inner segment of photoreceptors. Intra-cellular fatty acid transport proceeds through binding to FABP.

**Fig. 5:**
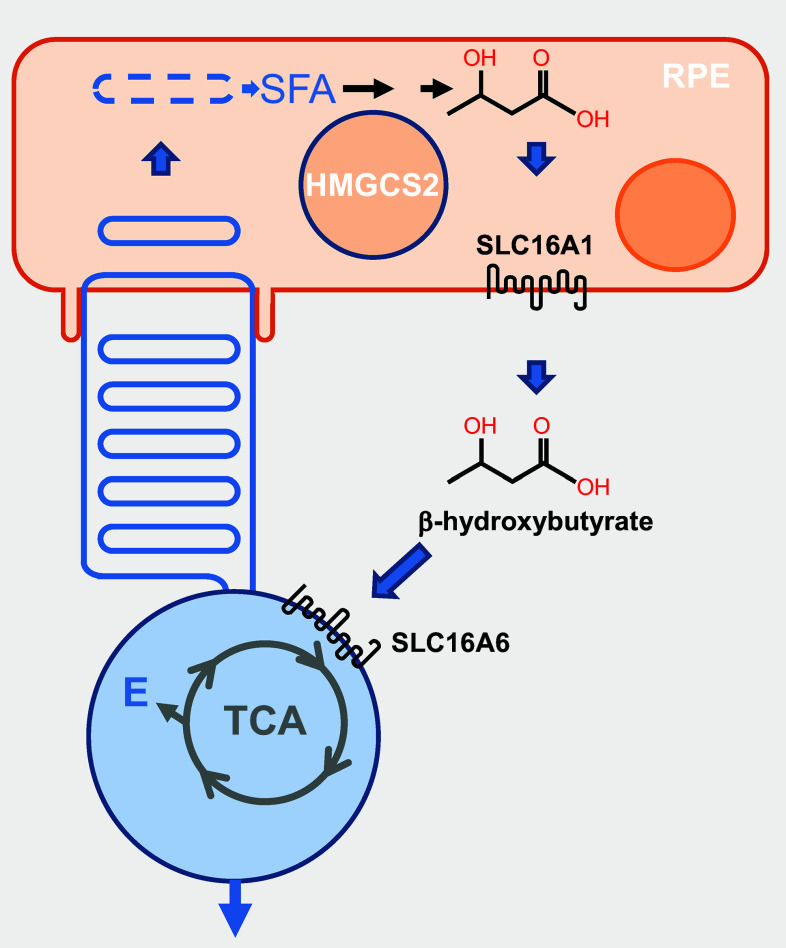
Recycling of saturated fatty acids by photoreceptors. *RPE* retinal pigmented epithelium, *SFA* saturated fatty acid, *HMGCS2* mitochondrial HMG-coenzyme A (CoA)-synthase 2, *SLC16A1* monocarboxylate transporter isoform 1, *SLC16A6* monocarboxylate transporter isoform 7, *TCA* tricarboxylic acid, *E* glutamic acid

Quite interestingly, a yet unknown fraction of the saturated fatty acids from photoreceptor outer segments is recycled to provide energy instead of structural components to photoreceptors. They are metabolized by RPE cells from the phagosome by the high levels of mitochondrial HMG-coenzyme A (CoA)-synthase 2 (HMGCS2) into ketone derivatives (C=O), then enzymatically processed into β-hydroxybutyrate by fatty acid β-oxidation pathway [22] (Fig. 5). β-Hydroxybutyrate is then released preferentially into the apical compartment through the monocarboxylate transporter isoform 1 (SLC16A1), facing photoreceptor cells that internalize it through the monocarboxylate transporter isoform 7 (SLC16A6). β-Hydroxybutyrate is oxidized by the tricarboxylic acid (TCA) cycle to produce energy and glutamate (E), a neurotransmitter involved in the vertical transmission of the signal from photoreceptors to bipolar cells [23]. The contribution of this cycle to the enrichment of photoreceptor outer segments into PUFAs is presently unknown.

## Renewing proteins of photoreceptor outer segments

Rhodopsin represents 80 % of total protein content in rod outer segments and a density of 25,000 molecule/mm^2^ on the disk membrane forming a supramolecular organization of tracks of rhodopsin dimers [24–26]. The pace of renewal of photoreceptor outer segment imposes that a high level of protein biosynthesis occurs on daily basis. Disk assembly at the base rod outer segment is estimated to be 80 disks per day, which requires the synthesis of ~1000 rhodopsin molecules per minute [27]. This requires efficient mechanism of transcription and translation. Integral transmembrane proteins and peripheral membrane proteins are then crossing the connecting cilium to reach photoreceptor outer segment. Transport of proteins and lipids through the cilium is mediated by the intra-flagellar transport (IFT) machinery.

## Natural history of cones and rods

As explained previously, in pulse-chase experiments, newly radiolabeled proteins migrate toward the connecting cilium and are incorporated into nascent disks of rod photoreceptors that move progressively toward the RPE [28]. In the cones, the radiolabeled proteins diffuse throughout the entire outer segment, because of the absence of disks; the outer segment membrane of cones is in direct continuation of the plasma membrane [29]. The difference in the morphology of the two classes of vertebrate photoreceptors was originally described by Max Schultze in 1866 [30]. In most vertebrates, vision is based on a dual system of photoreceptors. The rods are responsible for scotopic vision, in conditions of low luminosity, and the cones are responsible for photopic vision, in conditions of high luminosity, for color vision and high-acuity. Color vision is the process by which information is extracted from a visual stimulus based on its wavelength composition. It is based on differences in spectral sensitivities of visual pigments, or opsins. In general, these opsins are expressed in a specific type of photoreceptors, in accordance with the principle of one receptor–one neuron that applies to most sensory systems. The different groups of cone opsins are defined in terms of the spectral sensitivity: cluster S (blue, ultraviolet <440 nm), clusters M1 and M2 (440–510 nm), and cluster L (red >500 nm) [31]. Most mammals have a retina, in which rods predominate. Nonetheless, despite this predominance of photoreceptors designed for night vision, many mammals have developed a diurnal lifestyle, in which vision is essentially dependent on cone activity. This paradox of a rod-dominated retina in animals adapted to diurnal activity applies to humans and other primates. High-acuity vision, in particular, is dependent on the presence of the fovea, a specialized region in the centre of the retina constituted exclusively of cones in its most central part. The absence of M1 and M2 opsin genes in all sequenced mammalian genomes led to the hypothesis that the common ancestor of all mammals probably had a nocturnal lifestyle not requiring complex color vision. The selection pressure exerted on the first mammals by contemporary diurnal sauropsids forced primitive mammals to adopt a nocturnal lifestyle relying on scotopic vision and explains the loss of M1 and M2 opsin genes. The sudden extinction of the dinosaurs enabled mammals to colonize the vacated diurnal ecological niches, a mechanism known as nocturnal bottleneck [32]. Contemporary birds, which belong to the sauropsids, have a retina dominated by cones as presumably that of dinosaurs and contrarily to mammals [33, 34]. Primitive primates (prosimians) with a nocturnal life style, such as many lemurs, have only dichromatic vision. Humans and other old world apes (*Cercopithecidae*) have trichromatic vision, due to duplication of the L-opsin gene on the X chromosome. Humans thus have four visual pigments: rhodopsin (RHO), S-(OPN1SW, 425 nm), M-(OPN1MW, 530 nm), and L-opsins (OPN1LW, 560 nm), expressed by rods and the blue, green and red cones, respectively. Nevertheless, L-opsin duplication is not specific to *Cercopithecidae*, it also occurred in a family of *platyrrhines*, the howler monkeys (*Alouatta caraya*) [35]. This duplication is an independent event and more recent than that in the *Cercopithecidae*. The evolutionary history of color vision of primates illustrates the importance of the cone in the acquisition of complex behaviors.

The most ancient ciliary photoreceptors in *Cnidarians* (corals, sea anemones and jellyfishes) share with vertebrate cones a low sensitivity to light and are adapted primarily for diurnal vision [36]. This observation asks the question of the origin of the rods. A major step in the evolution of the vertebrate eye was the emergence of rods in addition to cones to produce a duplex retina [37]. In a duplex retina, rods are functional for dim light vision with great sensitivity, and when the light intensity increases, the rods are saturated and turned off, leaving the cones to function in bright light, greatly reducing energy required for vision. Functional rods evolved before the split between the jawed and jawless vertebrates. Sea lamprey (*Petromyzon marinus*) has two types of photoreceptors, the short and the long both with a cone-like morphology of outer segment, but the short photoreceptors have a single-photon sensitivity similar to that of rods in other vertebrates [38–40]. The typical rod outer segment morphology with segmented disks was acquired later during evolution, probably because it allows only the removal of the oldest macromolecules during outer segment disk shedding contrarily to cones. An increased metabolic rate, along with changes in energy allocation, was crucial in the evolution of human brain size and life history [41]. One could speculate that the reduction in energy requirement for vision had permitted energy allocations in favor of cephalization and cognitive functions during evolution of jawed vertebrates [42]. Citing Spinoza “Living organisms are designed with an ability to react emotionally to different objects and events”, we could propose that the light perception by photoreceptors and cognitive functions are parallel attributes of the same substance [43]. Intriguingly, in all conditions where the rods are destroyed, the cones degenerate secondarily. This is particularly well established in rod-dominated mammalian retina, but was also observed in retina with an equal proportion of rods and cones [44]. Ablation of rod photoreceptors in *Xenopus laevis* results in outer segment degeneration and cone cell death. In such retina, there are evidences that rods also need cones to survive [45]. A mutation in the cone-specific phosphodiesterase zebrafish gene (*pde6c*) triggers cone death followed by rod degeneration in areas of the retina that was originally rich in cones. In mouse models of rod-to-cone degeneration, it was proposed that glucose uptake by cones and/or its intra-cellular concentration in cones may be compromised [46]. The cones need the rods to survive [47].

## Cones got married to rods for life

**Fig. 6:**
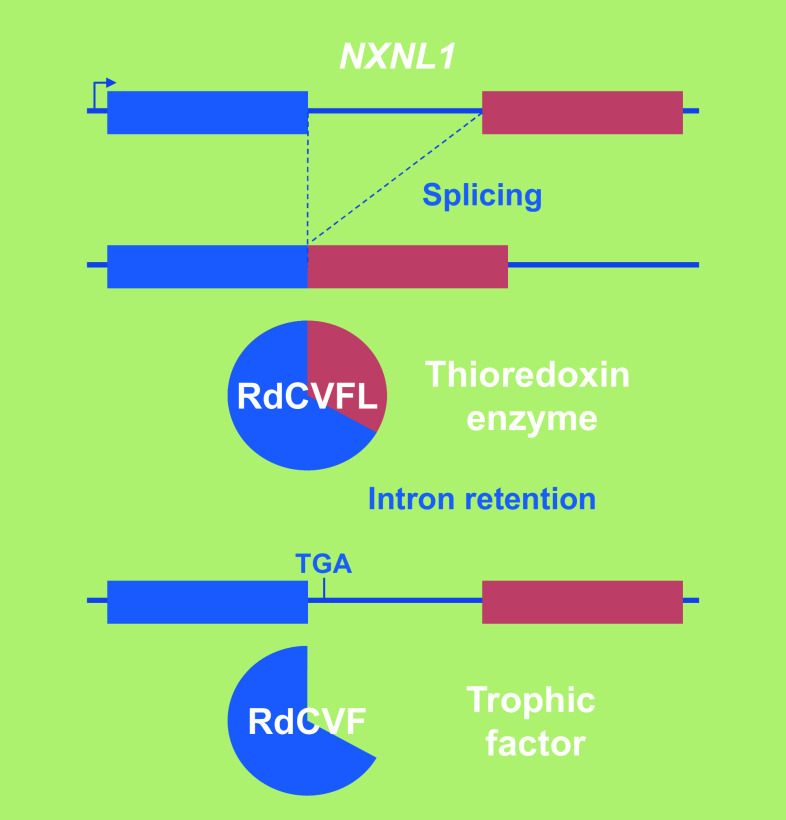
Genomic organization of the bifunctional gene nucleoredoxin-like 1. *NXNL1* nucleoredoxin-like 1, *RdCVFL* the thioredoxin enzyme rod-derived cone viability factor long, *RdCVF* the trophic factor rod-derived cone viability factor, *TGA* stop codon

In patients suffering from retinitis pigmentosa, the most common form of inherited retinal degeneration, the vision loss develops in two successive steps. Early in their adult life, these patients lose ability to see in dim light conditions that refers to a night vision lost and corresponds to the loss of function and degeneration of rod photoreceptors. This is felt as a minor handicap, especially in individuals affected by congenital stationary night blindness, another type of inherited retinal disease characterized exclusively by lack of rod function; in our current well-illuminated environment, these people retain an almost normal way of life [48]. For those patients affected with rod-cone dystrophy, the disease then progresses through another debilitating step resulting from loss of function and degeneration of the second class of photoreceptors, the cones that dominate at the centre of the retina. Cones represent only 3–5 % of all photoreceptors in most mammals, but their role for vision is essential. This secondary event leads to central vision loss and potentially complete blindness. Because the cones underlie all visual functions in lighted environment, cone rescue was deemed to be a clinically relevant target [49, 50]. Widespread cone death in the naturally occurring *rd1* mutant mouse, a model of retinitis pigmentosa, is well described [51]. The degeneration does not arise in this model through a mutation within cone photoreceptor cells, but as a result of a recessive mutation in the rod photoreceptor-specific cGMP phosphodiesterase-β subunit (PDE6B), and is consequently non-cell autonomous [52]. This mutation also leads to rod-cone degeneration in humans [53]. Grafting normal photoreceptors (97 % rods) into the eye of the rod-less *rd1* mouse before the cones degenerate exerts a positive effect on the host retina cones [54]. Co-culture studies demonstrated that such trophic effect on cone photoreceptors might be mediated through a diffusible factor [55]. Rod death in the first phase of the disease is triggered by the loss of expression of rod-derived cone viability factor (RdCVF), a truncated thioredoxin-like protein encoded by the nucleoredoxin-like-1 gene (*NXNL1*) [56, 57]. The inactivation of the *Nxnl1* gene in the mouse triggers an age-dependent loss of cones even with the potential complementation of its paralog *Nxnl2* [58–60]. RdCVF is a translation product made from an alternatively spliced mRNA encoding the exon 1 and retaining the following intron that contains an in-frame stop codon (Fig. 6). The other product (RdCVFL), made by splicing intron 1 of the *NXNL1* gene, is an active thioredoxin enzyme that protects rod and cone photoreceptors against photo-oxidative stress [61–64]. *NXNL2*, the paralog of *NXNL1*, also encodes for at least two proteins: RdCVF2, a trophic factor produced by the rods and active on cones, and the thioredoxin protein RdCVF2L by alternative splicing [60, 65]. The administration of RdCVF protein would restore rod-to-cone signaling preventing the secondary degeneration of cones and thus transforming retinitis pigmentosa in a type of night blindness associated with moderate visual impairment, independent from the causative mutations in any of the 60 known genes [66–68]. Injection of RdCVF in animal models of retinitis pigmentosa prevents the shortening of cone outer segments, which precedes cone loss [69]. The secondary degeneration of cones in retinitis pigmentosa patients occurs over a period of more than a decade with an average loss of 4 % of visual acuity per year [70]. Preventing the secondary loss of cone by the administration of RdCVF is medically rational, since most patients that consult an ophthalmologist have already lost most of the rods, while visual acuity is only reduced when 50 % of the cones become non-functional [71].

## Rods feed the cones

**Fig. 7:**
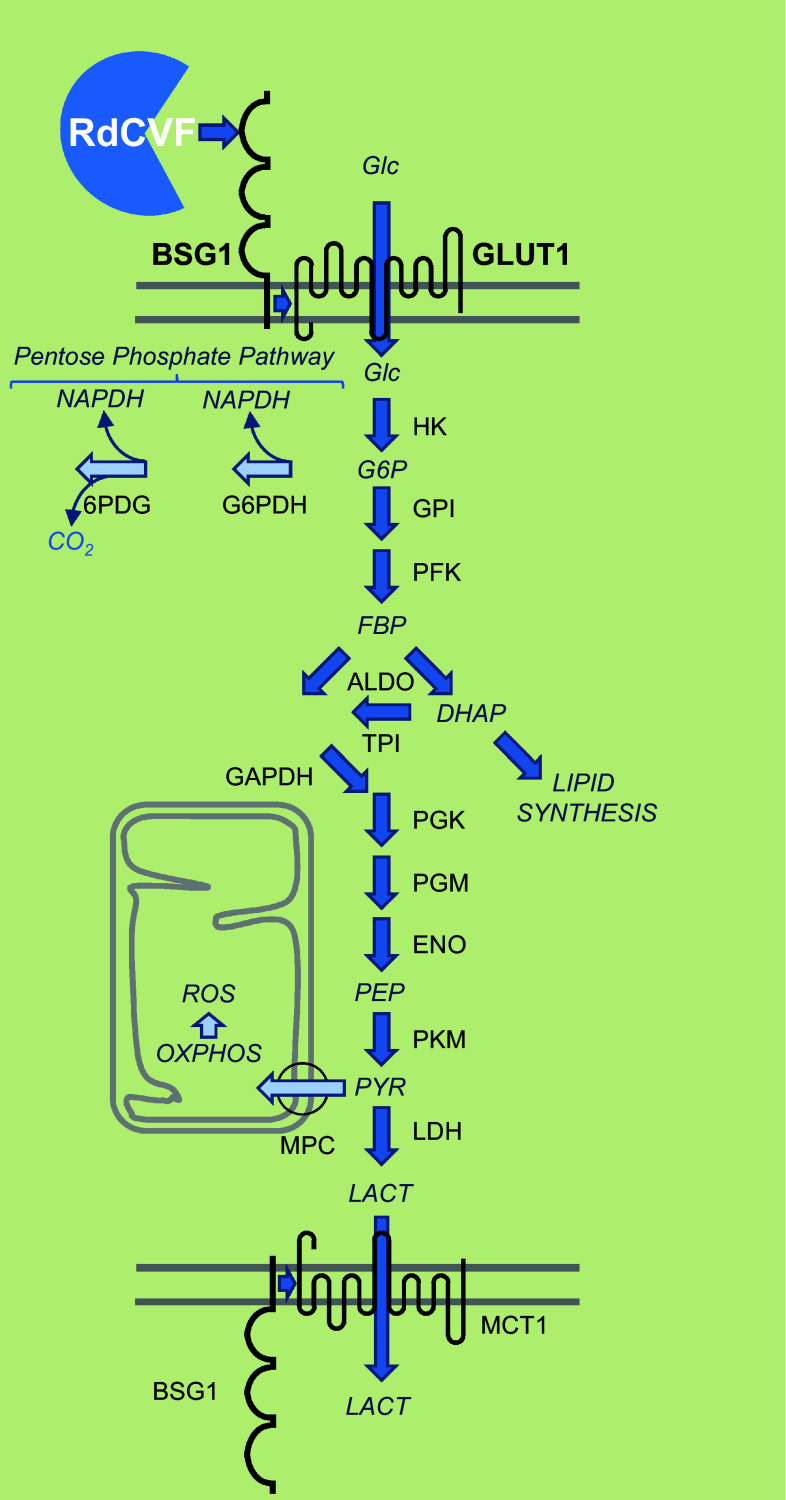
Metabolic signaling regulated by rod-derived cone viability factor. *Top-to-bottom* *RdCVF* rod-derived cone viability factor, *BSG1* basigin-1, *GLUT1* glucose transporter *SLC2A1*, *Glc* glucose, *G6P* glucose-6-phosphate, *FBP* fructose biphosphate, *DHAP* dihydroxyacetone phosphate, *PEP* phosphoenol pyruvate, *PYR* pyruvate, *LACT* lactate, *MPC* mitochondrial pyruvate carrier, *HK* hexokinase, *GPI* glucose-6-phosphate isomerase, *PFK* phosphofructokinase, *ALDO* aldolase, *TPI* triosephosphate isomerase, *PGK* phosphoglycerate kinase, *PGM* phosphoglycerate mutase, *ENO* enolase, *PKM* pyruvate kinase M, *LDH* lactate dehydrogenase, *MCT1* lactate transporter SLC16A, *NADPH* nicotinamide adenine dinucleotide phosphate, *G6PDH* glucose-6-phosphate dehydrogenase, *6PDG* 6-phosphogluconate dehydrogenase, *OXPHO* oxidative phosphorylation, *ROS* reactive oxygen species

RdCVF binds basigin-1 (BSG1), its cell-surface receptor on cones [72]. BSG1 is a photoreceptor-specific alternative spliced isoform of the *BSG* gene, which has three extracellular immunoglobulin (Ig) domains contrarily to BSG2, the other product of the same gene more broadly expressed that possesses only two Ig domains [73]. RdCVF binds to BSG1, but not to BSG2. BSG1 forms a complex with the glucose transporter GLUT1 at the cone surface, whose transport activity is increased by RdCVF binding. Glucose is metabolized by cones via aerobic glycolysis to produce metabolites necessary for renewing cone outer segments (Fig. 7). Cone survival relies on the ability of RdCVF to stimulate aerobic glycolysis [74, 75]. GLUT1 catalyses the rate-limiting step in supplying cells of the central nervous system. The trophic effect of RdCVF via aerobic glycolysis is thus mediated by a three proteins complex-containing RdCVF, basigin-1, and GLUT1. This is distinct from the effect of insulin on cones that is mediated by the insulin receptor [46, 76–79]. GLUT1 exists in equilibrium between homodimeric and homotetrameric forms [80]. Each subunit of GLUT1 contains an extracellular disulfide bridge (C_347_ and C_421_) that stabilizes the tetrameric structure and thereby accelerates transport function by increasing the Vmax of transport and decreasing the dissociation constant, Km [81]. GLUT1 reduction causes GLUT1 tetramers to dissociate into dimers. RdCVF binding to basigin-1 may somehow displace the equilibrium toward the tetramer, accelerating GLUT1 transport function and stimulating glucose uptake by cones acting in this scheme as an allosteric modulator of GLUT1. When Otto Warburg described aerobic glycolysis as a hallmark of cancer cells in 1922, he also identified the retina as an exception to this observation even if he thought that could be an artifact of tissue preparation [82]. Aerobic glycolysis in mammalian retina is providing carbohydrates metabolite used for the daily renewal of 10 % of the outer segments of photoreceptors [83]. Similarly, cancer cells proliferate and rely on the production of carbohydrate intermediates at a high rate [84, 85]. Metabolic reprogramming of cancer cells to Warburg effect is a primary transformation occurring prior to the activation of proto-oncogenes and inactivation of tumor suppressor genes [86]. Pyruvate is at a crucial metabolic branch point [87]. When transported into mitochondria by the mitochondrial pyruvate carrier, a heterodimer composed of MPC1 and MPC2, pyruvate is oxidized to Acetyl-CoA by the multi-subunit pyruvate dehydrogenase complex localized in the mitochondrial matrix. Acetyl-CoA then enters the TCA cycle, where it is further oxidized. Following glycolysis and oxidative phosphorylation, each fully oxidized molecule of glucose to CO_2_ produces 30 molecules of ATP. Alternatively, pyruvate can also be reduced to lactate in the cytosol by lactate dehydrogenase. This reaction allows the regeneration of NAD^+^ from the nicotinamide adenine dinucleotide (NADH) that is produced by glycolysis. Aerobic glycolysis departs from oxidative phosphorylation in the glycolytic part of the reaction by the involvement of hexokinase 2 (HK2) instead of hexokinase 1 and pyruvate kinase isoform M2 (PKM2) instead of PKM1. Hexokinase 2 (HK2) is highly activated in cancer cells and is located on the outer membrane of mitochondria [88]. Both HK2 and PKM2 are expressed preferentially by photoreceptors [72, 89–91]. *Hk2* expression is increased during the maturation of photoreceptor in the mouse retina. RdCVF does not activate the expression of HK2, nor does it promote a switch from oxidative phosphorylation to aerobic glycolysis [72]. Hexokinases are responsible for a rate-limiting step of glycolysis phosphorylating glucose to glucose-6-phosphate (G6P), which is maintained in the cytoplasm. G6P is the branch point for proceeding to glycolysis or the pentose phosphate pathway (PPP) (Fig. 7). The PPP shunt is the major contributor of reducing equivalents in the form of reduced nicotinamide adenine dinucleotide phosphate (NADPH). Pyruvate kinases catalyze an ATP-generating step of glycolysis, in which phosphoenolpyruvate (PEP) is converted to pyruvate. Pyruvate kinase exists in two M isoforms, differentiated by alternative splicing of exons 9 and 10, which in PKM2 codes for a specific allosteric pocket, absent in PKM1 that allows the binding of the activating glycolytic intermediate, fructose-1,6-bisphosphate (FBP) [92]. PKM2 controls the final step of glycolysis, and its regulation serves to integrate intra-cellular signaling inputs with the metabolic state of the cell. Down-regulation of PKM2 activity and up-regulation of other enzymes committing glucose to glycolysis will lead to the accumulation of phosphorylated glycolytic intermediates that spill into branching biosynthetic pathways, as the production of glycerol-3-phosphate from dihydroxyacetone phosphate (DHAP) or the production of NADPH through the pentose phosphate pathway [93] (Fig. 7). The carbon flux must be diverted, since if each six-carbon glucose is entirely transformed producing two molecules of three-carbon lactate, no carbon would be incorporated from glucose into phospholipids of the cone outer segments. Injection of glucose in a pig model of retinitis pigmentosa was sufficient to promote cone outer segment regrowth, which is consistent with the mode of action of RdCVF [94].

For retinitis pigmentosa patients, in the midterm of their disease, the regrowth of cone outer segments, which ultimately will reverse the disease by restoring central vision can be theoretically obtained by stimulating the rate of aerobic glycolysis by increased glucose entry and by increasing the expression and/or the activity of key glycolytic enzymes [72, 77]. Cone outer segment regrowth is a real possibility, as it was observed in patients suffering from acute idiopathic blind spot enlargement and after retinal detachment [95–97].

## Antioxidants versus redox signaling

According to the free radical theory of aging, first proposed by Harman in 1956 [98], free radicals are continuously produced in the cell as a product of aerobic life and induce oxidative damage while aging [99]. Caloric restriction, which lower glucose metabolism, is the only unequivocally effective scheme to extend life span significantly for most organisms and is known to increased resistance to various oxidative stresses in many animals [100]. More than 90 % of total cellular oxygen is reduced to water stepwise via electron carriers of the mitochondrial respiratory chain [101]. Presently, it is virtually impossible to identify which specific respiratory complex or mitochondrial enzyme is producing reactive oxygen species by leakage [102]. Reactive nitrogen species (RNS) are produced when the superoxide ion O_2_^·−^ reacts with nitric oxide (NO) produced by NO synthases (NOS1–3). To avoid the damage of macromolecules by ROS and RNS, proper redox conditions must be maintained within the intra-cellular environment. Therefore, aerobic organisms have developed several antioxidant systems. Antioxidant molecules, such as uric acid, glutathione (GSH), and vitamins C and E, scavenge ROS and RNS to prevent oxidative damage. Antioxidant enzymes detoxify ROS/RNS into less reactive species. Paradoxically, the protective role of antioxidants has been challenged by the observation that antioxidants prevent health-promoting effects of physical exercise in humans [103].

**Fig. 8:**
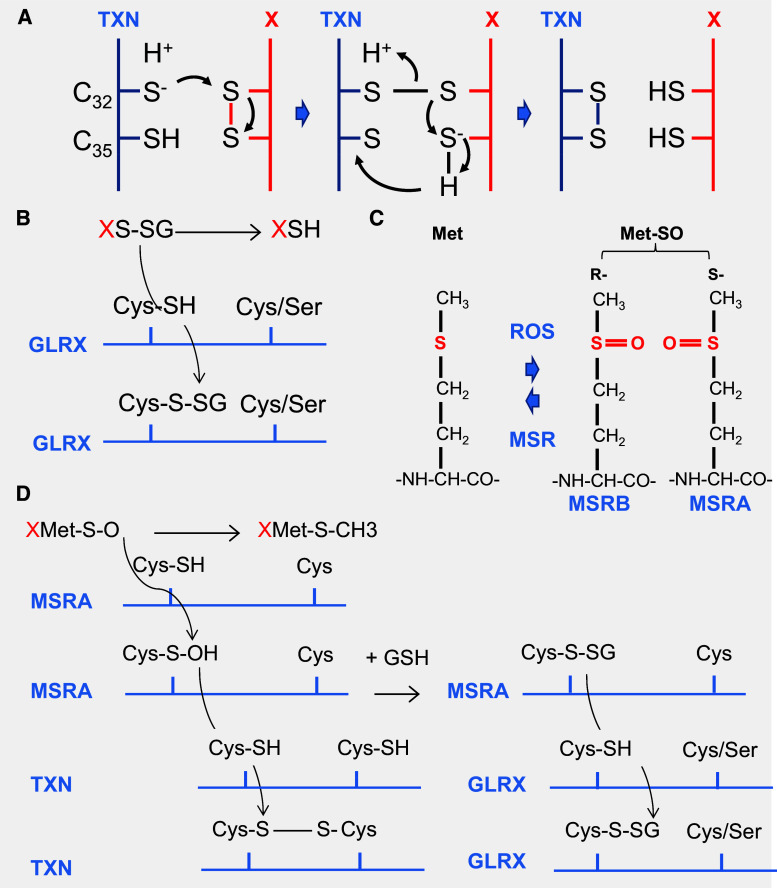
Thioredoxin/glutaredoxin system. **a** The oxidoreduction reaction between the thioredoxin (TXN) and its substrate (X protein). Reduced thioredoxin TXN-SH_2_ binds to a target protein X via its hydrophobic surface area. Nucleophilic attack by the thiolate of Cys_32_ results in the formation of a transient mixed disulfide, which is followed by a nucleophilic attack of the deprotonated Cys_35_ generating oxidized TXN-S2 and the reduced protein, X-SH_2_. **b** Deglutathionylation reaction by glutaredoxin (GLRX). **c** The oxidation of methionine generates a diastereomeric mixture of two stereoisomers methionine *S*-sulfoxide and methionine *R*-sulfoxide. *Met-SO* methionine sulfoxide* MSRA* and * MSRB* methionine sulfoxide reductase A and B, *ROS* reactive oxygen species. **d** Methionine sulfoxide reductase A reaction. *MSRA* methionine sulfoxide reductase A, *TXN* thioredoxin, *GLRX* glutaredoxin reductase, *GSH* glutathione

Alternatively, repairing enzymes reduces the oxidized groups in macromolecules. Importantly, the reversible oxidation of the thiol group in cysteines and methionines is the rational of redox signaling [104]. Cysteine is a rarely used amino acid that accounts for about 2 % of the amino acids in eukaryotic proteins. ROS and RNS can induce redox signals by means of oxidative modifications of cysteine residues. The large, polarizable sulfur atom in a thiol group is electron-rich and highly nucleophilic; hence, cysteines can undergo a broad range of chemical reactions. The sulfhydryl group (C–SH) is in equilibrium with C–S^−^ and to the disulfide (S–S), can be oxidized by ROS to sulfenic (C–SOH), sulfinic (SO_2_H), and sulfonic (SO_3_H), or *S*-nitrosylated by RNS to C–SNO, and finally, in the presence of glutathione (GSH), *S*-thiolated to C–S–SG [6]. Sulfonic acid modifications, the most oxidized form of the thiol group, are irreversible and are thus deleterious oxidative damage, much as HNE adducts [105]. Cysteines differ in their reactivity properties depending of the protein microenvironment, and not all cysteines are susceptible to modifications. Most, but not all, of these modifications are reversible through reduction catalyzed by oxidoreductases, such as thioredoxins (TXN1 and 2), glutaredoxins (GLRX1-3 and 5), and sulfiredoxin (SRXN1). The prototype of thioredoxin proteins, TXN1, is a 12 kDa protein with a redox active conserved disulfide/dithiol group C_32_GPC_35_ (Fig. 8a). Reduced TXN1 catalyses the reduction of disulfide bounds in many proteins, and oxidized TXN1 is reversibly reduced by the action of thioredoxin reductases (TXNRD1–3) and NADPH [106]. TXN is secreted from cells by a hitherto unknown mechanism that is not dependent on a signal peptide, as for RdCVF [107]. Transgenic mice overexpressing human TXN1 have a statistically significant increase in life span [108]. Under oxidative conditions, heterologous disulfides can be formed non-enzymatically between proteins and the tripeptide glutathione (GSH), one of the most prevalent and important thiol buffers in the cell. Reduced GSH is oxidized to GSSG or in protein-S–SG (XS–SG). This reaction is termed *S*-glutathionylation [109]. *S*-Glutathionylation is a protection of protein thiols under oxidative conditions, since it can be reversed [110]. It prevents the sequential oxidation of thiol groups to sulfenic, sulfinic, and sulfonic acids; the latter is irreparably damaged [111]. Deglutathionylation is catalyzed by glutaredoxins (GLRX) through a monothiol reaction that depends only on the N-terminal active site cysteine residue (Fig. 8b). GSSG is reduced by the mitochondrial glutathione reductase (GSR). GSR requires riboflavin (vitamin B2) in the flavin adenine dinucleotide (FAD) coenzyme form to perform the reduction of GSH: FADH_2_ + GSSG > FAD + GSH [112].

Methionine is also sensitive to non-enzymatic oxidation by ROS. Methionine sulfoxide (Met-SO) can be further oxidized to sulfone (Met-SO_2_) by an irreversible oxidation. The oxidation of methionine generates a diastereomeric mixture of two stereoisomers methionine, *S*-sulfoxide and methionine *R*-sulfoxide, due to the asymmetry of sulfur atom that is repaired by methionine sulfoxide reductases: MSRA and MSRB1-3, respectively (Fig. 8c). Most MSR possess two cysteine residues (2-Cys-MSR), a catalytic cysteine and a recycling cysteine. For both MSRA and MSRB, the catalytic cysteine of the MSR first attacks the sulfur atom of Met-SO in a nucleophilic reaction. The catalytic cysteine forms a disulfide bond with the recycling cysteine, MSR is oxidized, and Met-SO is reduced to Met in the reaction (Fig. 8d). The recycling of MSR involves either TXN or GLRX.

## The pentose phosphate pathway links glucose metabolism to redox signaling

**Fig. 9:**
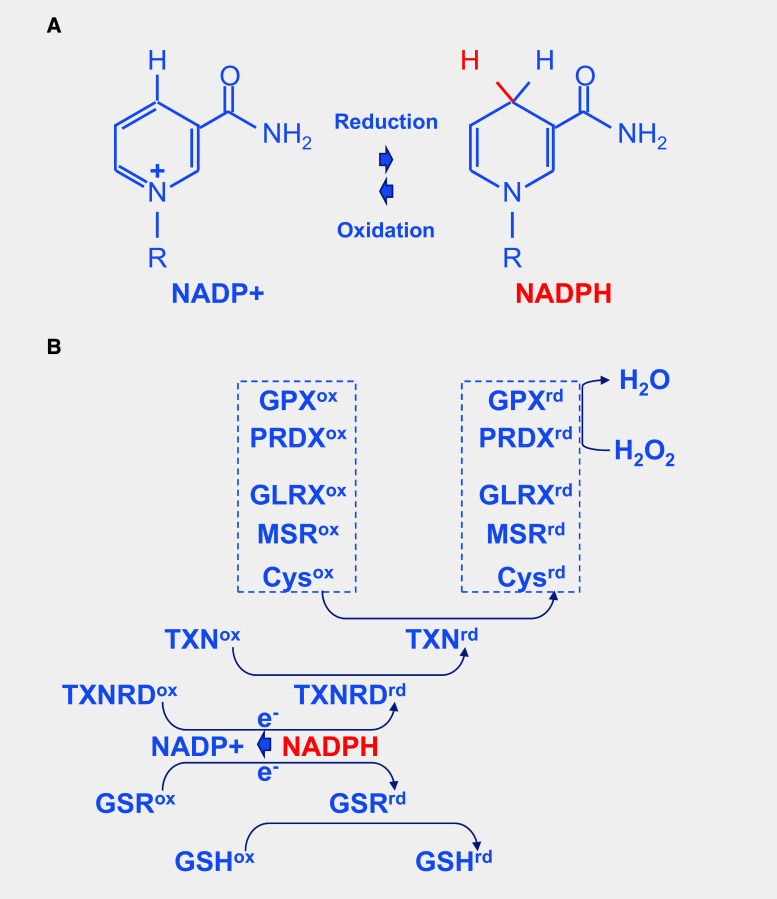
Redox power is regulated by the production of NADPH by the pentose phosphate pathway. **a** Oxidoreduction of NADP^+^ and NADPH. **b** Schematic drawing of the thioredoxin/glutaredoxin system. *TXNRD* thioredoxin reductase, *GSR* glutathione reductase, *GSH* glutathione, *TXN* thioredoxin, *Cys* cysteine, *MSR* methionine sulfoxide reductase, *GLRX* glutaredoxin, *PRDX* peroxiredoxin, *GPX* glutathione peroxidase. The suffix *ox* and *rd* represent the oxidized and reduced forms, respectively

**Fig. 10:**
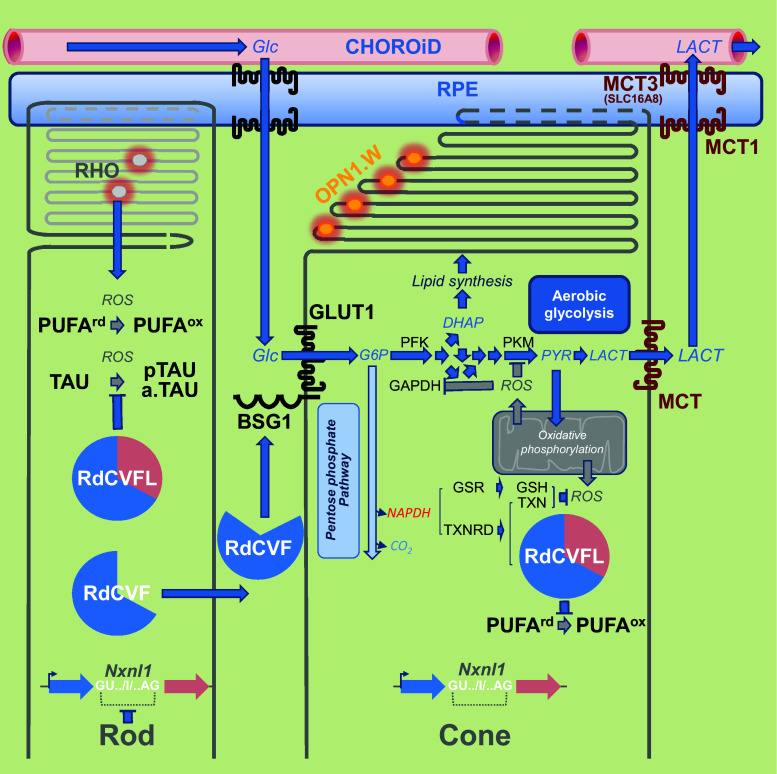
Metabolic and redox signaling of the *NXNL1* gene products. Rods produce the thioredoxin RdCVFL and the trophic factor RdCVF by alternative splicing. Cones exclusively produce the thioredoxin RdCVFL. RPE: retinal pigmented epithelium. *Left to right* *RHO* rhodopsin, *PUFA* polyunsaturated fatty acid, *TAU* microtubule-associated protein *TAU*, *BSG1* basigin-1, *GLUT1* glucose transporter *SLC2A1*, *Glc* glucose, *G6P* glucose-6-phsphate, *DHAP* dihydroxyacetone phosphate, *PYR* pyruvate, *LACT* lactate, *PFK* phosphofructokinase, *PKM* pyruvate kinase M, *GAPDH* glyceraldehyde-3-phosphate dehydrogenase, *ROS* reactive oxygen species, *NADPH* nicotinamide adenine dinucleotide phosphate, *TXNRD* thioredoxin reductase, *GSR* glutathione reductase, *TXN* thioredoxin, *GSH* glutathione, *MCT1* lactate transporter 1 (SLC16A1), *MCT3* lactate transporter 3 (SLC16A8). The suffix p and a. represent, respectively, the phosphorylated and the aggregated forms. Framing the coding intron I of the *NXNL1* genes, GU.. and ..AG, is the splicing donor and acceptor sites, respectively. The suffix *ox* and suffix *rd* represent the oxidized and reduced forms, respectively

The reduction of NADP^+^ to NADPH is particularly important, because it provides reducing power for most antioxidant and redox regulatory enzymes controlling cell redox homeostasis. Nicotinamide is derived from ATP [113] (Fig. 9a). In response to oxidative stress, glucose metabolism is diverted from energy formation to reductive biosynthesis [114]. Glyceraldehyde-3-phosphate dehydrogenase (GAPDH) harbors a strictly conserved catalytic cysteine, which is susceptible to a variety of thiol modifications, including inhibitory but reversible *S*-glutathionylation [115]. Oxidation of PKM at cysteine 358 (C358) stops the production of pyruvate and results in the accumulation of phosphoenol pyruvate (PEP) [93]. PEP, an allosteric inhibitor of triose phosphate isomerase (TPI), interrupts glycolysis, which produces an elevation of the intra-cellular concentration of glucose-6-phosphate (G6P). G6P, produced by the action of hexokinases on glucose entry to the cell via glucose transporters, is diverted to produce ribulose-5-phosphate (Ru5P) by the oxidative part of the pentose phosphate pathway, the enzymatic steps catalyzed by glucose-6-phosphate dehydrogenase (G6PDH) and 6-phosphogluconate dehydrogenase (6PGD), the later producing also CO_2_ (Fig. 7). Both G6PDH and PGD use NADP^+^ as a co-factor that is reduced to NADPH during the reaction. The electrons derived from NADPH are transferred to the glutathione and thioredoxin systems via thioredoxins reductases (TXNRD1–3) and glutathione reductase (GSR), respectively, to reduce ROS and to reduce oxidized proteins (Fig. 9b). Thus, the cellular antioxidant machineries are maintained with energy provided by glucose catabolism through NADPH-mediated electron transport [116]. G6PDH activity is regulated by the NADP^+^/NADPH ratio, NADPH inhibits its activity, whereas NADP^+^ is required for its proper active conformation [114]. In the non-oxidative part of the pentose phosphate pathway, Ru5P is converted to ribose-5-phosphate (R5P) by ribulose-5-phosphate isomerase (RPIA), and R5P might reenter the glycolytic pathway when converted in fructose-6-phosphate (F6P) or glyceraldehyde-3-phosphate (G3P) [117]. Increased flux of glucose through the pentose phosphate pathways can have a neuroprotective function [118]. The conversion of G6P to Ru5P generates two moles of NADPH and one of CO_2_, so that under conditions of excess oxidative stress requiring the maximal amount of NADPH, G6P is completely oxidized to CO_2_ by six complete cycles. The complete oxidation of glucose by cells to CO_2_ is likely to be detrimental to the organism, but in non-pathological conditions, the production of redox power through NADPH should balance the negative effect of oxidation of glycolytic enzymes, such as GAPDH and PKM and restore redox homeostasis (Fig. 10).

## Oxidative stress promotes retinal diseases

Epidemiological studies have suggested that elderly patients who consumed diets rich in antioxidants throughout their life are less likely to be afflicted with age-related macular degeneration (AMD) [119]. In animal models, the endogenous antioxidant molecule taurine and its derivatives are able to prevent photoreceptor degeneration following exposure to damaging light [120, 121]. There is also an attenuation of retinal photo-oxidative damage in thioredoxin transgenic mice [122]. There is an additional cause of oxidative stress to photoreceptors in the course of normal life or in inherited diseases with rod-specific mutations. A problem with the choroidal circulation lies in inefficient self-regulatory mechanisms that fail to meet the demands of the tissue it serves. One consequence is that when the oxygen consumption of the photoreceptors falls, oxygen tension in the outer retina rises sharply [123]. Consequently, the retinal oxygen pressure reaches values that approximate hyperoxia. Moreover, cone death can be delayed by reducing oxidative stress during disease [124, 125].

The disruption of the rod-derived cone viability gene leads to photoreceptor dysfunction and susceptibility to oxidative stress [58]. Cone function is normal in young *Nxnl1* ^−*/*−^ mice, but deteriorates month-by-month as the mice age, indicating that the gene is involved in the late (but not early) developmental stages, or aging. Specifically, RdCVFL interacts with the microtubule-associated protein TAU (TAU) and prevents its oxidation in vitro. It also prevents its phosphorylation and aggregation in the retina [58, 126]. RdCVFL protects rod photoreceptors against photo-oxidative damage [64] and reduces the oxidation of polyunsaturated fatty acids induced by photoreceptor degeneration in the *rd10* mouse [66]. RdCVFL is normally expressed by cones as well as rods in the mouse retina, while the trophic factor RdCVF is specifically expressed by rods: alternative splicing of the *Nxnl1* gene is restricted to rods [127]. Differences between splicing pattern between rods and cones were revealed by the study of the *PRPH2* gene [128]. The cones and their outer segments composed of polyunsaturated fatty acids are damaged by reactive oxygen species produced from leakage of the respiratory chain. In the absence of *Nxnl1*, the retina shows the signs of lipid peroxidation, as HNE adducts [58]. After selective recombination of the *Nxnl1* gene in the cones, the retina also displayed signs of oxidative damage. Nevertheless, in the retina, the thiol-oxidoreductase activity of RdCVFL depends on its cycling between a reduced and an oxidized status as any other thioredoxin. Once oxidized, RdCVFL must be reduced by thioredoxin reductases [106]. The co-factor of thioredoxin reductases, NADPH, is produced mostly from glucose through the pentose phosphate pathway. In that respect, in cones, RdCVF likely acts upstream of RdCVFL by providing reducing power (Figs. 7 and 10). Reduced RdCVFL could reestablish aerobic glycolysis in cones after oxidative stress, and we have identified a potential interaction between RdCVFL and PKM [126]. One could speculate that in patients suffering from retinitis pigmentosa after the loss of expression of RdCVF produced by rods, cones become non-functional and die within several years as a result of the loss RdCVFL expression and subsequent oxidative damage in cones. Thus, a therapy aimed at preventing secondary cone degeneration should be pursued using both RdCVF and RdCVFL.

## Perspectives opened by metabolic and redox signaling in the retina

The retina is a biological model system that was at the origin of many breakthroughs in biology and the metabolic and redox signaling revealed by the study of the mechanism of secondary degeneration of cones in retinitis pigmentosa might be part of these scientific founding principles. Nevertheless, we are perfectly aware that our report will become obsolete in the near future, since the research on the retina is a very active field. We simply intend to draw here the big scene that could both incorporate the numerous new findings that can be anticipated and evolve accordingly.

## Abbreviations

6PGD: 6-Phosphogluconate dehydrogenase
ADIPOR1: Adiponectin receptor protein 1
AMD: Age-related macular degeneration
BSG1: Basigin-1
BSG2: Basigin-2
DHA: Docosahexaenoic acid
DHAP: Dihydroxyacetone phosphate
F6P: Fructose-6-phosphate
FABP: Fatty acid-binding proteins
FAD: Flavin adenine dinucleotide
FBP: Fructose-1,6-bisphosphate
G3P: Glyceraldehyde-3-phosphate
G6P: Glucose-6-phosphate
G6PDH: Glucose-6-phosphate dehydrogenase
GAPDH: Glyceraldehyde-3-phosphate dehydrogenase
GLRX: Glutaredoxin
GLUT1: Facilitated glucose transporter SLC2A1
GPX: Glutathione peroxidase
GSH: Glutathione
GSR: Glutathione reductase
HK: Hexokinase
HMGCS2: HMG-coenzyme A-synthase 2
HNE: Hydroxy-2-nonenal
IFT: Intra-flagellar transport
Ig: Immunoglobulin domain
IPM: Inter-photoreceptor matrix
IRBP: Inter-photoreceptor retinoid-binding protein
MCT1: Monocarboxylate transporter 1 SLC16A1
MCT3: Monocarboxylate transporter 3 SLC16A8
MDA: Malondialdehyde
Met-SO: Methionine sulfoxide
MFSD2A: Sodium-dependent lysophosphatidylcholine symporter 1
MSR: Methionine sulfoxide reductases
NADH: Nicotinamide adenine dinucleotide
NADPH: Nicotinamide adenine dinucleotide phosphate
NO: Nitric oxide
NXNL1: Nucleoredoxin-like-1 gene
OPN1LW: Long-wave-sensitive opsin 1
OPN1MW: Medium-wave-sensitive opsin 1
OPN1SW: Short-wave-sensitive opsin 1
PDE6B: Phosphodiesterase-b subunit
PEP: Phosphoenolpyruvate
PKM: Pyruvate kinase isoform M
PPP: Pentose phosphate pathway
PRDX: Peroxiredoxin
PRPH2: Peripherin-2
PUFA: Polyunsaturated fatty acids
R5P: Ribose-5-phosphate
rd1: Retinal degeneration 1 mouse
RdCVF: Rod-derived cone viability factor
RdCVF2: Rod-derived cone viability factor 2
RdCVF2L: Rod-derived cone viability factor 2 long
RdCVFL: Rod-derived cone viability factor long
RHO: Rhodopsin
RNS: Reactive nitrogen species
ROS: Reactive oxygen species
RPE: Retinal pigmented epithelium
RPIA: Ribulose-5-phosphate isomerase
Ru5P: Ribulose-5-phosphate
SLC: Solute carrier family of transporters
SLC16A: Monocarboxylate transporter family 16
SRXN: Sulfiredoxin
TAU: Microtubule-associated protein TAU
TCA: Tricarboxylic acid
TPI: Triose phosphate isomerase
TXN: Thioredoxin
TXNRD: Thioredoxin reductase
